# Cultural differences are reflected in variables associated with carer burden in FTD: a comparison study between India and Australia

**DOI:** 10.1590/S1980-57642013DN70100016

**Published:** 2013

**Authors:** Shailaja Mekala, Suvarna Alladi, Kammammettu Chandrasekar, Safiya Fathima, Claire M.O.'Connor, Colleen McKinnon, Michael Hornberger, Olivier Piguet, John R. Hodges, Eneida Mioshi

**Affiliations:** 1 Nizam's Institute of Medical Sciences, Hyderabad, India.; 2 Asha Psychiatric Hospital, Hyderabad, India.; 3 Neuroscience Research Australia, Sydney, Australia.; 4 Western Sydney Local Health District, Sydney, Australia.; 5 Neuroscience Research Australia, Sydney, Australia. School of Medical Sciences, University of New South Wales, Sydney, Australia.

**Keywords:** carer burden, caregiver burden, carer depression, carer anxiety, carer stress, dementia severity

## Abstract

There is great need to understand variables behind carer burden, especially in FTD. Carer burden is a complex construct, and its factors are likely to vary depending on the type of dementia, carer characteristics and cultural background.

**OBJECTIVE:**

The present study aimed to compare profiles and severity of carer burden, depression, anxiety and stress in carers of FTD patients in India in comparison to Australia; to investigate which carer variables are associated with carer burden in each country.

**METHODS:**

Data of 138 participants (69 dyads of carers-patients) from India and Australia (India, n=31; Australia, n=38). Carer burden was assessed with the short Zarit Burden Inventory; carer depression, anxiety and stress were measured with the Depression, Anxiety and Stress-21. Dementia severity was determined with the Frontotemporal Dementia Rating Scale (FTD-FRS), and a range of demographic variables regarding the carer and patient were also obtained.

**RESULTS:**

Overall, levels of carer burden were not significantly different across India and Australia, despite more hours delivering care and higher dementia severity in India. Variables associated with burden, however, differed between countries, with carer depression, anxiety and stress strongly associated with burden in India. By contrast, depression, stress, and dementia severity were associated with burden in Australia.

**CONCLUSION:**

This study demonstrated that variables associated with carer burden in FTD differ between cultures. Consequently, cultural considerations should be taken into account when planning for interventions to reduce burden. This study suggests that addressing carers' skills and coping mechanisms are likely to result in more efficacious outcomes than targeting patient symptoms alone.

## Abstract (pt)

Há uma grande necessidade de se entender as variáveis por trás da sobrecarga do cuidador, especialmente em DFT. A sobrecarga é um construto complexo e os fatores provavelmente estão ligados ao tipo de demência, características do cuidador e origens culturais.

**OBJETIVO:**

O presente estudo objetivou comparar perfis e gravidade da sobrecarga, depressão, ansiedade e estresse nos cuidadores dos pacientes com DFT da Índia em comparação aos da Austrália; investigar que variáveis do cuidador estão associadas à sobrecarga em cada país.

**MÉTODOS:**

Dados de 138 participantes (69 pares cuidadores-pacientes) da Índia e Austrália (Índia, n=31) e Austrália (n=38). A sobrecarga do cuidador foi avaliada através da versão curta do Inventário de Sobrecarga de Zarit; depressão, ansiedade e estresse do cuidador através com o *Depression, Anxiety and Stress-21*. A gravidade da demência foi determinada com a *Frontotemporal Dementia Rating Scale* (FTD-FRS), e uma gama de variáveis demográficas do cuidador e do paciente foram também obtidas.

**RESULTADOS:**

De modo geral os níveis de sobrecarga do cuidador não foram significativamente diferentes entre Índia e Austrália, apesar do maior tempo despendido no cuidado e gravidade da demência na Índia. As variáveis associadas à sobrecarga, todavia, diferiram entre os países, com depressão do cuidador, ansiedade e estresse fortemente associados com sobrecarga na Índia. Em contraste, depressão, estresse e gravidade da demência foram associados à sobrecarga na Austrália.

**CONCLUSÃO:**

Este estudo demonstrou que variáveis associadas à sobrecarga do cuidador na DFT difere entre culturas. Consequentemente, aspectos culturais devem ser levados em consideração quando se planeja intervenções para redução da sobrecarga. Este estudo sugere que programas direcionados às habilidades e meios de se lidar com a situação dos cuidadores são provavelmente mais eficazes do que aqueles só aos sintomas do paciente.

## INTRODUCTION

Carer burden is a multifaceted and complex construct mediated by a number of variables and their interactions. In frontotemporal dementia (FTD), a recent study has shown that disease severity is the main factor contributing to high levels of reported carer burden.^1^ Other studies have shown that carer-based variables such as depression of the carer is also very relevant.^2^ Finally, patient-related variables such as concurrent cognitive deficits and age at disease onset have also been recently recognised.^3^ The question in examining which variables contribute to carer burden in FTD is important given the particularly high level of carer burden in FTD compared to Alzheimer's disease.^1,4,5^

Most of the studies of burden of care in FTD, however, have been conducted in Western countries,^1-3,6-8^ with cross-cultural differences virtually unexplored. It is very likely that carers' needs are likely to reflect the environment that they live in, and the amount of support (emotional, services, cultural) to which they have access to, as well as their perception of what is available to them.^9^

In India, dementia is largely unrecognised as a disease and a great proportion of the population (including health professionals)^10^ is not aware (or not willing to consider?) of the impact of dementia and their devastating symptoms in individuals and their families. Cognitive decline is accepted as part of the normal ageing process in a large proportion of the Indian population, and has been for centuries. This is commonly called "turned 60".^10^ This term is used regardless of age of onset of cognitive decline, and still prevails: not surprisingly, it makes recognition of dementia as a disease very difficult by the general population. As a result, dementia-related symptoms and their consequences on an individual's work, social participation, and leisure, are "naturally" absorbed by the family structures (and their paid carers). In addition, the limited number of services and support programs available offers no alternative choice for families. With the rising numbers of people with dementia in India,^11^ and increasing burden of disease on families and society, there is an imminent need to understand specific factors that are associated with the burden. Carer burden is under recognised in India. High levels of carer strain have been reported among Indian carers which correlated with factors such as severity of dementia, behavioural problems in patients, time spent caring and a lack of support from services.^12-16^

This raises several cross-cultural questions, such as whether a fatalistic approach to dementia symptoms, as in India, would impact on carer burden differently compared to a country where dementia is widely recognised as a disease, such as Australia. How would FTD affect carers in India, given its socially challenging symptoms and early onset? A few studies have investigated burden of carers of Alzheimer's disease dementia patients in India,^12,14,16^ and none has investigated this issue in FTD or across countries.

The aims of this study were: [1] to compare profiles and severity of carer burden, depression, anxiety and stress in carers of FTD patients in India in comparison to Australia; [2] to investigate which carer variables are associated with carer burden in each country.

## METHODS

**Participants.** This study included data from 138 participants (69 dyads of carers-patients) from India and Australia (India, n=31; Australia, n=38). Data from India were collected from December 2009 to May 2012 at the Memory Clinic, Nizam's Institute of Medical Sciences, in Hyderabad. Data for all Australian dyads were collected at first visit at the FTD clinical research group Frontier, in Sydney (December 2007 to May 2011). Data were collected by local researchers in each centre, all of them with clinical professional background (neuropsychologists, behavioural neurologists and occupational therapists). The Hyderabad team was trained by the senior author (EM) on measures that were not previously used in their centre. Carer instruments were self-complete, and were sent to the spouses to be completed at home (Sydney), or were completed while waiting for the research appointment (Hyderabad). The FRS was given via an interview at the research centre, and the FRS takes, on average, 15 minutes to be administered.

Patients were diagnosed with FTD according to current consensus criteria.^17,18^ Patients were excluded if exhibiting major depressive illness, or if carers were not a relative of the patient. All carers were primary carers of the person with dementia.

At the time of the study, all Indian patients were community dwellers; all but one Australian patient were community dwellers (97.4%). Patients from both countries were matched for length of symptoms, as shown in Table 1, but not for dementia severity. All caregivers and/or patients consented to the study and Ethics approval was obtained from ethics committees in India and Australia.

**Table 1:** Demographic characteristics of carers from India and Australia and patient dementia characteristics.

	Indian carers (n=31)	Australian carers (n=38)	India vs Australia*
Age	54.7 (11.1)	57.7 (13.2)	n.s.
Education (years)	13.6 (4.2 )	13.3 (2.9)	n.s.
Sex of carer, % females	61.3	78.9	n.s.
Number of people helping carer regularly	1.9 (0.8)	1.5 (0.7)	n.s.
Number of hours caring for the patient (per week)	101.4 (66.3)	64.2 (57.5)	p <0.01
Length of symptoms (years)	2.6 (1.9)	3.3 (1.9)	n.s.
Disease severity (FRS)	-1.418 (severe)	0.035 (moderate)	p<0.05

* t test

**Instruments.** *Carer burden: Zarit Burden Inventory (ZBI)* - Carer burden was measured using the short Zarit Burden Inventory,^19^ which asks carers to rate their feelings towards care in terms of frequency (self-complete). The 12 questions are summed up to a maximum score of 48. High scores denote increased burden, with a suggested cutoff score of 17 indicating clinically significant burden.^19^

*Carer depression, anxiety and stress: DASS-21* - The Depression, Anxiety and Stress Scale 21 (DASS 21)^20^ was applied to evaluate depression, anxiety and stress of the carers. This tool is self-complete, with a maximum score of 42. Existing normative data suggest cutoff scores of 10 and above reflecting significant depression, 8 and above indicative of significant anxiety, and 15 and above for significant stress.^21^

*Dementia severity* - Stage of dementia was determined with the Frontotemporal Dementia Rating Scale (FTD-FRS).^22^ This scale has been developed specifically for FTD and is widely used internationally. The FRS is administered via an interview with the informant, and yields 6 disease stages: very mild, mild, moderate, severe, very severe and profound. Questions are adjusted for the individual pre-morbid functioning to avoid bias in the score.

**Statistical analysis.** Demographic data were compared across countries via student t tests. Tests of normality (Kolmogorov-Smirnoff) showed that a number of variables of interest were not normally distributed. For this reason, a non-parametric approach was chosen, with Mann-Whitney tests for comparison between countries, and Spearman correlations (with Bonferroni corrections, p<0.01) for multiple comparisons between variables associated with burden (ZBI). Chi square tests were used to compare proportions of carers (between countries) above cut-off in the ZBI and DASS. Alpha was set at 0.05 unless otherwise stated.

## RESULTS

**Carer demographics.** The majority of carers were female, in both countries. Carers were matched for age, number of years in full time education, type of relationship, and number of people helping the carer in looking after the patient. Number of hours providing direct care was greater for Indian carers (Table 1).

**Figure 1:**
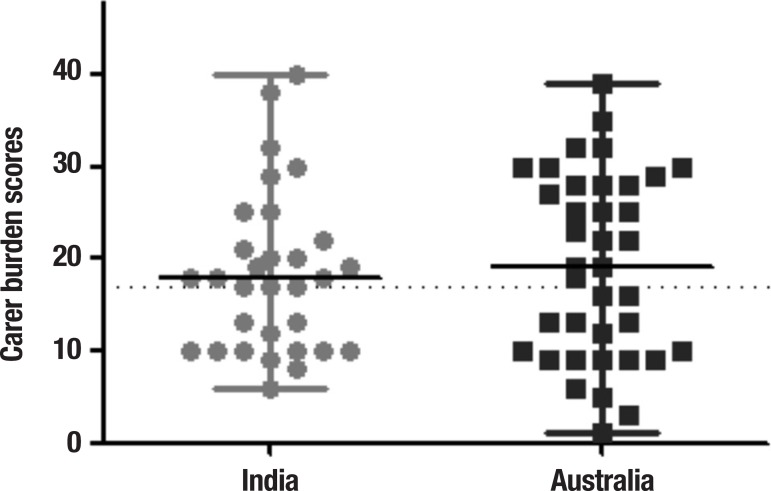
Carer burden scores on the Zarit Burden Inventory in India and Australia represented in medians and interquartile ranges. Dotted line represents clinical cut off for high burden. Mann-Whitney tests, p<0.05.

**Carer burden: Zarit Burden Inventory (ZBI).** The burden of care reported by carers in both countries was not statistically different as shown in Figure 1. In India, 61.3% of carers reported high levels of burden; in Australia the proportion was 55.3%.

**Figure 2:**
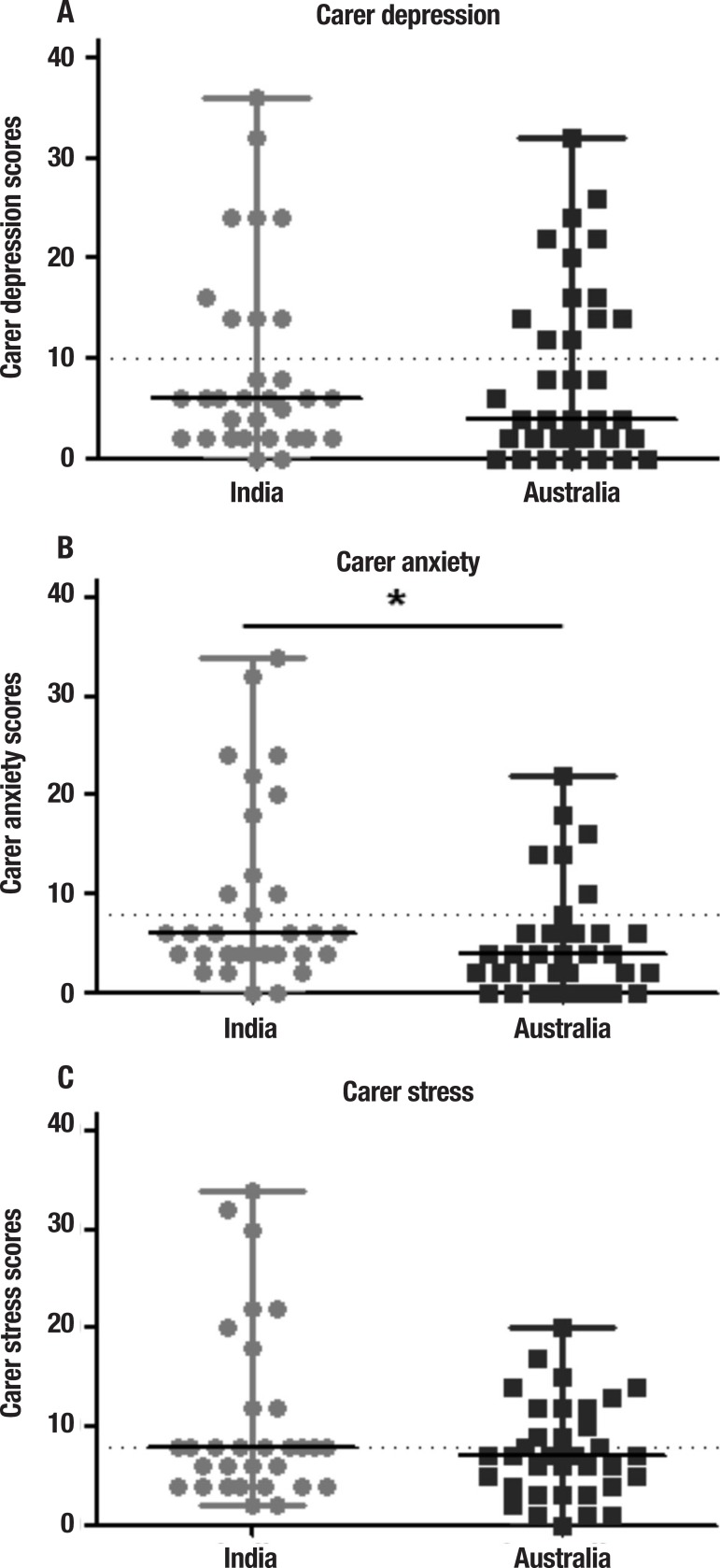
Comparison of scores on carer [A] depression, [B] anxiety and [C] stress on the Depression, Anxiety and Stress-21, between India and Australia. Dotted lines represent clinical cut off. Mann-Whitney tests, p<0.05.

**Carer depression, anxiety and stress: DASS-21.** No significant differences were found between Indian and Australian carers in their levels of depression (Figure 2A) and stress (Figure 2C). In contrast, however, carers in India reported significantly higher levels of anxiety compared to Australian carers (p<0.05) (Figure 2B).

In terms of clinical cut-offs, in India, high levels of anxiety were present in 35.5% of carers; depressive symptoms were present in 29%; high levels of stress in 22.6%. For Australian carers, depressive symptoms were common (36% of carers were above cut off), followed by 20% of carers reporting high levels of anxiety and only 9.1% reporting high levels of stress. No significant differences between the two countries were found in the proportions of carers above cut-offs for depression, anxiety and stress (all p values >0.05).

*Which variables are associated with carer burden?* To examine potential differences between variables influencing the burden of Indian and Australian carers, correlations between variables were performed in each country. The main variables of interest were disease severity^1^ and depression,^2^ based on previous studies in FTD. In addition, given that no studies in carer burden in FTD in India have been published, we also examined the potential roles of carer anxiety and stress.

**Figure 3:**
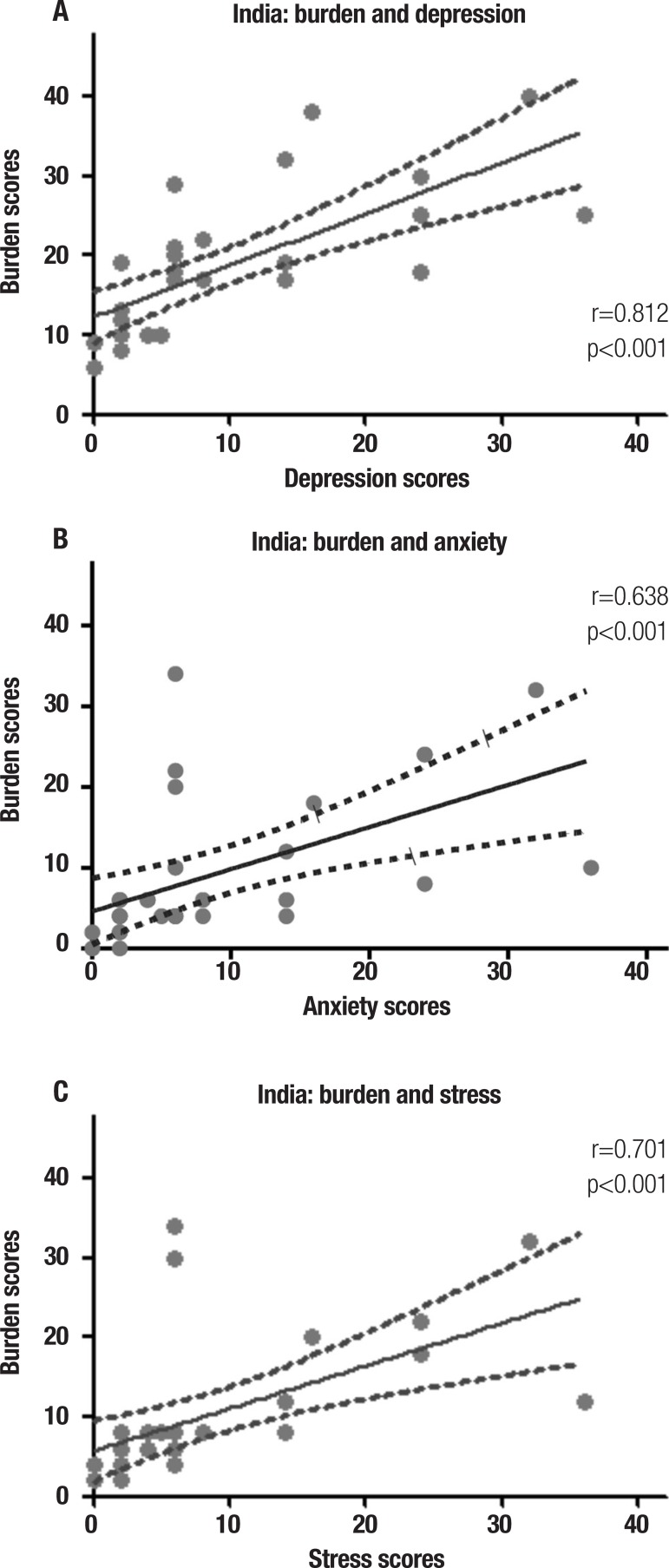
Variables associated with carer burden in the Indian sample: depression, anxiety and stress.

For Indian carers, burden was not associated with dementia severity (p=0.785). However, carer burden was significantly associated with depression (r=0.812, p<0.001), anxiety (r=0.638, p<0.001) and stress (r= 0.701, p<0.001) (Figure 3).

In the Australian sample, carer burden was significantly associated with stress (p<0.001) and depression (p<0.001), but not with dementia severity.

## DISCUSSION

This study is the first to compare carer burden in FTD in two countries with vastly different cultures. Our findings revealed that levels of carer burden were similar between India and Australia, despite higher levels of dementia severity and greater number of hours providing direct care in the Indian sample compared to the Australian sample. Additionally, this study demonstrated that variables associated with carer burden differed across countries. In India, depression, anxiety and stress were all significantly associated with carer burden. In Australia, depression and stress were strongly associated with burden, and dementia severity to a lesser degree in comparison with the other variables.

The strong association between carer burden and disease severity in FTD that we previously reported^1^ was again observed given that both studies utilised data from the same participants from the Australian sample. Despite caring for a more impaired group of patients, Indian carers reported the same levels of stress as Australian carers. Acceptance of the dementia process as part of normal ageing could be a major factor in this, resulting in higher tolerance levels to dementia in India;^10^ however, this factor was not directly investigated in the current study. Alternatively, sample sizes might have also played a role in the results reported here, given that this sample was relatively smaller than that in the first study.

Reported levels of anxiety were greater in Indian than in Australian carers, while depression and stress levels were similar in the two samples. This finding suggests that even though the acceptance of dementia might "protect" Indian carers from higher levels of carer burden, in comparison to Australia, this protection does not extend to their levels of anxiety. It is plausible that Indian carers report more anxiety because the symptoms they observe and experience are regarded as "normal" in the ageing process,^23^ leaving them with little room to address and obtain skills to reduce them. Moreover, cultural variation in expression of anxiety and depression exist. Somatic symptoms of anxiety rather than depressive feelings are more often in Asian and Indian cultures^24^ and this is likely to have influenced higher anxiety scores. Even if participants in this study involved patients properly diagnosed and carers who were well informed, it is likely that this sample was still influenced by the cultural attitudes that surround them, such as cultural obligations towards the care of the ill and elderly, the indivisibility of older patients with the younger family members in India and the resulting strain associated with the care of someone with dementia.^25^ The predominantly home based care for dementia patients in India,^26^ and a lack of adequate supportive health care services may be additional factors that play a role in this difference.

The examination of variables associated with burden revealed novel findings. In the Indian sample, depression, anxiety and stress were all strongly associated with burden. These findings suggest that burden is dependent on carer rather than patient variables, such as dementia severity. A previous study has also reported that number of hours devoted to caregiving is also an important factor^27^ in predicting higher burden, which surprisingly might not have had a direct contribution to Indian carers. In this study, burden in Australian carers was also associated with carer-based variables, but dementia severity still played a role in high levels of carer burden, as previously demonstrated. This finding suggests that interventions addressing carer coping skills might have a greater impact than those targeting dementia specific symptoms, especially in India.

This study had some limitations. Because of the sample sizes in both countries, a limited number of variables and statistical analyses were used. Future studies would benefit from including other variables not examined here, such as use of services, and previous caring role experience.

In summary, levels of carer burden in FTD were similar across India and Australia, despite greater dementia severity in Indian patients, and greater number of hours delivering care. Variables associated with carer burden were mostly dependent on the carer, especially in India, revealing the need to skill carers and providing them with information which will clarify and validate what they go through. Carer interventions need to take into account the multitude of variables behind carer burden, including cultural background of carer, for more efficacious results.
